# Small risk of developing symptomatic tick-borne diseases following a tick bite in the Netherlands

**DOI:** 10.1186/1756-3305-4-17

**Published:** 2011-02-10

**Authors:** Ellen Tijsse-Klasen, Jac J Jacobs, Arno Swart, Manoj Fonville, Johan H Reimerink, Afke H Brandenburg, Joke WB van der Giessen, Agnetha Hofhuis, Hein Sprong

**Affiliations:** 1 Laboratory for Zoonoses and Environmental Microbiology, Centre for Infectious Disease Control Netherlands, National Institute for Public Health and Environment (RIVM), Bilthoven, the Netherlands; 2 Laboratory for Infectious Diseases and Screening, Centre for Infectious Disease Control Netherlands, National Institute for Public Health and Environment (RIVM), Bilthoven, the Netherlands; 3 Epidemiology and Surveillance Unit, Centre for Infectious Disease Control Netherlands, National Institute for Public Health and Environment (RIVM), Bilthoven, the Netherlands; 4 General practice Ballum, the Netherlands; 5 Laboratorium voor de Volksgezondheid in Friesland, Leeuwarden, the Netherlands

## Abstract

**Background:**

In The Netherlands, the incidence of Lyme borreliosis is on the rise. Besides its causative agent, *Borrelia burgdorferi *s.l., other potential pathogens like *Rickettsia*, *Babesia *and *Ehrlichia *species are present in *Ixodes ricinus *ticks. The risk of disease associated with these microorganisms after tick-bites remains, however, largely unclear. A prospective study was performed to investigate how many persons with tick-bites develop localized or systemic symptoms and whether these are associated with tick-borne microorganisms.

**Results:**

In total, 297 *Ixodes ricinus *ticks were collected from 246 study participants who consulted a general practitioner on the island of Ameland for tick bites. Ticks were subjected to PCR to detect DNA of *Borrelia burgdorferi s.l.*, *Rickettsia *spp., *Babesia *spp. or *Ehrlichia/Anaplasma *spp.. Sixteen percent of the collected ticks were positive for *Borrelia burgdorferi s.l*., 19% for *Rickettsia *spp., 12% for *Ehrlichia*/*Anaplasma *spp. and 10% for *Babesia *spp.. At least six months after the tick bite, study participants were interviewed on symptoms by means of a standard questionnaire. 14 out of 193 participants (8.3%) reported reddening at the bite site and 6 participants (4.1%) reported systemic symptoms. No association between symptoms and tick-borne microorganisms was found. Attachment duration ≥24 h was positively associated with reddening at the bite site and systemic symptoms. Using logistic regression techniques, reddening was positively correlated with presence of *Borrelia afzelii*, and having 'any symptoms' was positively associated with attachment duration.

**Conclusion:**

The risk of contracting acute Lyme borreliosis, rickettsiosis, babesiosis or ehrlichiosis from a single tick bite was <1% in this study population.

## Background

The most prevalent and widespread vector-borne disease of humans and animals in the northern hemisphere is Lyme borreliosis. Early detection of Lyme borreliosis is crucial, as antibiotics are most effective at this stage, preventing the development severe sequelae [1]. Over the last decade, the incidence of Lyme borreliosis has increased significantly in Europe, with up to 16 and 21 cases per 10,000 individuals reported in Scandinavia and Slovenia, respectively [2]. A periodical retrospective study under general practitioners in The Netherlands has shown a continuing and strong increase in general practitioner (GP) consultations for erythema migrans and hospital admissions in the past 15 years with 22000 cases in 2009. The most straightforward explanation for this increase is the concomitant increase in the number of GP consultations for tick bites [3,4]. Although direct evidence is lacking, the factors responsible for this increase are most probably a combination of higher tick numbers and intensified human recreational behaviour, leading to an increased exposure of the population to tick bites.

The same tick species transmitting the etiologic agents of Lyme disease in Europe, *Ixodes ricinus*, also serves as vector of pathogens causing tick-borne encephalitis, babesiosis, several forms of rickettsioses and anaplasmoses. Incidences and public health risks of tick-borne diseases other than Lyme borreliosis are largely unknown in The Netherlands, but also in other countries. Although Dutch ticks have been shown to have a high prevalence of *Rickettsia helvetica *and can contain *Babesia*, so far no endemic disease cases in humans have been observed for these microorganisms [5]. *R. helvetica *is a intracellular bacterium that is suspected of causing acute perimyocarditis, unexplained febrile illness and sarcoidosis [6-11]. Various *Babesia *species are known to cause disease in humans and animals with *Babesia divergens *probably being the most important human pathogenic species in Europe but one European case of *B. microti *infection has been reported as well [12-15]. *Anaplasma *and *Ehrlichia *have also been found in Dutch ticks and a few human cases have been reported in the Netherlands [16,17].

Not only transmitted pathogens but also the tick itself can lead to health impairments. Ticks secrete a complex mixture of bio-active compounds, mainly proteins, during the blood meal [18-20]. These can have local or systemic toxic effects or induce an immune response. Individuals rarely react with intense anaphylaxis but milder allergic reactions are probably more common and easily overlooked [21-23].

The risk of developing Lyme borreliosis or any other tick-borne disease after a tick bite depends on many unrelated factors, including the tick species, the number of pathogens per tick, the site and duration of the tick bite, the (genetic) constitution of the pathogen and the individual susceptibility to infection [24-26]. Many, if not all, of these factors may vary geographically and in time. Several prospective studies have estimated the risk of developing Lyme borreliosis following a tick bite, but not in The Netherlands, and rarely for other tick-borne diseases such as spotted fever rickettsiosis, babesiosis, and anaplasmosis [27-30]. Data on Borrelia infection on patients and ticks from the years 2004-2006 were previously reported [31].

Field studies between 1989 and 1993 on the Dutch island of Ameland reported a *B. burgdorferi s.l. *prevalence in ticks of all life stages (n = 521) of 24% (95% CI: 20.7-28.0%) [32,33], which led to an increased awareness of Lyme disease on the island. To estimate the risk of developing symptoms following tick bites and to investigate whether this risk is associated to specific tick-borne pathogens a study was initiated. Patients who consulted one GP on Ameland for a tick bite were invited to participate. They were asked to fill out a questionnaire on the day they visited the GP and were approached again several months later. The ticks were tested for *Borrelia burgdorferi s.l.*., *Rickettsia *spp*., Babesia *spp. and *Ehrlichia*/*Anaplasma *spp..

## Materials and methods

### Study participants

Between January 2004 and December 2008 ticks were collected from patients with one or more tick bites that consulted the GP in the village of Ballum on Ameland (53°44'41N, 5°68'43E). Ballum is the smallest village on Ameland with only about 370 citizens but about 500000 tourists visit this village yearly. This is represented in the composition of the study population of which only 20% was a resident of the island. Patients or their guardians were asked for an informed consent for testing the tick for various microorganisms and for collecting data via questionnaires or interviews. In a first questionnaire data were collected concerning, amongst others, the number of tick bites and duration of tick attachment. Approximately six months after the first visit to the GP patients were contacted by phone and interviewed. Some of the patients where only reached after 12 to 18 months. This second interview aimed at identifying possible symptoms related to the tick bite including local redness or erythema migrans and systemic symptoms as fever, malaise, palpitations, joint problems, or neurological symptoms, and if so whether they consulted a GP for this symptoms.

### Removal and analysis of ticks

Ticks were carefully removed by the general practitioner and were immersed in 70% ethanol or a lysis buffer (0,5% sodium dodecyl sulfate; 100 mM tris-(hydroxymethyl) aminomethane; 10 mM ethylenediaminetetraacetic acid; 10 mM NaCl; pH: 8,3; 0,5 mg/ml protease K) and sent to the laboratory. Ticks stored in ethanol were determined to species level and DNA was extracted as described earlier [31]. Tick DNA extracts were analyzed by polymerase chain reaction (PCR) followed by reverse line blotting (RLB), as described elsewhere [34]. Primers and probes are described in Table 1.

**Table 1:** Primers and probes used in this study for PCR and RLB.

Name	Sequence (5' - 3')	Type	Target	Species	Reference
5S borSeq	GAGTTCGCGGGAGAGTAGGTTATTGCC ^(1)^	Primer	23S-5S IGS	*B. burgdorferi *sensu lato	[38]
23S borSeq	TCAGGGTACTTAGATGGTTCACTTCC	Primer	23S-5S IGS	*B. burgdorferi *sensu lato	[38]
A-borsl1	CTTTGACCATATTTTTATCTTCCA	Probe	23S-5S IGS	*B. burgdorferi *sensu lato	[39]
A-borsl2	CTTCCATCTCTATTTAGCCAATTT	Probe	23S-5S IGS	*B. burgdorferi *sensu lato	[38]
A-borsl3	TATTTTTATCTTCCATCTCTATTTT	Probe	23S-5S IGS	*B. burgdorferi *sensu lato	[38]
B31-A-s.stricto	AACACCAATATTTAAAAAACATAA	Probe	23S-5S IGS	*B. burgdorferi *sensu stricto	[39]
Ga2-garinii	AACATGAACATCTAAAAACATAAA	Probe	23S-5S IGS	*B. garinii*	[39]
Vs46lN2afzelii	AACATTTAAAAAATAAATTCAAGG	Probe	23S-5S IGS	*B. afzelii*	[39]
VsII62 val	CATTAAAAAAATATAAAAAATAAATTTAAGG	Probe	23S-5S IGS	*B. valaisiana*	[39]
A-Ruski	GAATAAAACATTCAAATAATATAAAC	Probe	23S-5S IGS	*B. afzelii (variant ruski)*	[40]
A-LusiP	CAAAAAAATGAACATTTAAAAAC	Probe	23S-5S IGS	*B. lusitaniae*	[41]

B-GA1b	CGGGATCCCGAGTTTGCCGGGACTTCTTCT ^(1)^	Primer	*16SrRNA*	*Ehrlichia*/*Anaplasma*	[42]
16S8Fe	GGAATTCAGAGTTGGATCMTGGYTCAG	Primer	*16SrRNA*	*Eubacteria*	[43]
Ehr-all	TTATCGCTATTAGATGAGCC	Probe	*16SrRNA*	*Anaplasma *genus	[42]
A-HGE	GCTATAAAGAATAGTTAGTGG	Probe	*16SrRNA*	HGE agent	[42]
A-Eqph	TTGCTATAAAGAATAATTAGTGG	Probe	*16SrRNA*	*A. phagocytophilum*	[42]
A-dHGE	GCTATGAAGAATAGTTAGTG	Probe	*16SrRNA*	HGE agent (variant)	[42]
A-dPh	TTGCTATGAAGAATAATTAGT	Probe	*16SrRNA*	*A. phagocytophilum variant*	[38]
A-E.Schot	GCTGTAGTTTACTATGGGTA	Probe	*16SrRNA*	*A. schotti *(variant)	[42]
A-murisT	AGCTATAGGTTTGCTATTAGT	Probe	*16SrRNA*	*E. muris *T variant	[40]
A-Chaff	ACCTTTTGGTTATAAATAATTGTTA	Probe	*16SrRNA*	*E. chaffeensis*	[42]
A-can	TCTGGCTATAGGAAATTGTTA	Probe	*16SrRNA*	*E. canis*	[42]
A-Wolbach	CTACCAAGGCAATGATCTA	Probe	*16SrRNA*	*Wolbachia*	[38]

Rick-16S rev	ACTCACTCGGTATTGCTGGA ^(1)^	Primer	*16SrRNA*	*Rickettsia *genus	[41]
Rick-16S for	AACGCTATCGGTATGCTTAACA	Primer	*16SrRNA*	*Rickettsia *genus	[41]
A-Rickall	TTTAGAAATAAAAGCTAATACCG	Probe	*16SrRNA*	*Rickettsia *genus	[41]
A-Rhelv2	GCTAATACCATATATTCTCTATG	Probe	*16SrRNA*	*R. helvetica*	[41]
A-Rconor	CTTGCTCCAGTTAGTTAGT	Probe	*16SrRNA*	*R. conorii*	[41]
A-16SRickIRS	GTATATTCTCTACGGAAAAAA	Probe	*16SrRNA*	*Rickettsia *IRS3	[41]
A-RProwaz	CGGATTAACTAGAGCTCGCT	Probe	*16SrRNA*	*Rickettsia prowazekii*	[34]
A-RTyphi	CGGATTAATTAGAGCTTGCT	Probe	*16SrRNA*	*Rickettsia typhi*	[34]
A-NonHelv A	AATACCGTATATTCTCTACGGA	Probe	*16SrRNA*	Non*- Rickettsia helvetica*	[34]
A-NonHelv B	AATACCGTATATTCTCTGCGGA	Probe	*16SrRNA*	Non*- Rickettsia helvetica*	[34]

BATH-Rn	TAAGAATTTCACCTCTGACAGTTA ^(1)^	Primer	*18SrRNA*	*Babesia *genus	[44]
BATH-Fn	ACACAGGGAGGTAGTGACAAG	Primer	*18SrRNA*	*Babesia *genus	[44]
Catch all 2	GTAATGGTTAATAGGARCRGTT	Probe	*18SrRNA*	*Babesia *genus	[44]
Ba-div	GTTAATATTGACTAATGTCGAG	Probe	*18SrRNA*	*B. divergens*	[45]
Ba-mic 1	CCGAACGTTATTTTATTGATTT	Probe	*18SrRNA*	*B. microti*	[34]
Ba-mot	GCTTGCTTTTTTGTTACTTTG	Probe	*18SrRNA*	*B. motasi*	[44]
Ba-mic 2	GRCTTGGCATCWTCTGGA	Probe	*18SrRNA*	*B. microti*	[44]
Ba-EU1	CTGCGTTATCGAGTTATTG	Probe	*18SrRNA*	*B. EU1*	[34]

Probes were 5'-amino-labeled. ^(1^^) ^Reverse primer 5'-labeled with biotine tetraethyleneglycol

### Statistics

Outcomes were defined as redness on the site of the tick bite, and/or any systemic symptoms such as fever, malaise or pain. Risk factors that were investigated included positive PCR/RLB results for one of the micro-organisms (*Borrelia*, *Rickettsia*, *Babesia *or *Ehrlichia*/*Anaplasma*) in the ticks, any of these (= any micro-organism), duration (<24 h, between 24 h and 48 h, >48 h) and number of tick bites.

Firstly, we tested for all possible outcome - risk factor combinations, the strength of association (null hypothesis of unity odds ratio, i.e. no association). To this end, contingency tables were constructed and Fisher's exact tests were performed for each combination. All calculations were performed using R 2.11.1, using the 'Epi' package v1.1.17. For outcomes and binary risk factors (i.e. absence-presence of microorganisms), risk ratios and their 95% confidence intervals were computed. For non-binary risk factors categories were defined. Furthermore, we calculated the exact p-value for the hypothesis of unity odds-ratio.

For purposes of logistic regression, it is convenient to know what factors are associated at the 70% significance level [35]. Significant factors and categorical data (number of bites, number of days the tick was attached) were included in backwards logistic regressions for each outcome variable. Any combination of microorganism and days of attachment was also tested in the logistic regression.

## Results

Ticks were collected from 246 study participants. In total 297 ticks were removed ranging from 1 to 18 ticks per individual with an average of 1.2 ticks per individual. All ticks that were identified to species level were *Ixodes ricinus*. Life stages of 236 ticks could be determined microscopically. Of these ticks, 65 (28%) were adults, 133 (56%) were nymphs and 38 (16%) were larvae. At least 53 participants were bitten by adult ticks, 96 by nymphs and 16 by larvae. Two hundred-ninety-four ticks were tested for *Borrelia burgdorferi s.l., Rickettsia *spp. and *Babesia *spp., 286 ticks were also tested for *Ehrlichia/Anaplasma *spp.. One-hundred-ninety-three (78.5%) participants were reached for a second interview, 51 participants were lost to follow-up. For epidemiological analysis only data of the responding participants were used.

**Figure 1:**
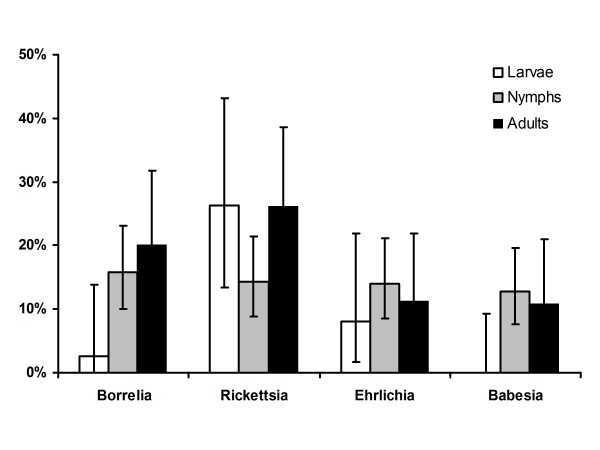
**Prevalence of microorganisms in different tick stages**. Ticks of different life stages were collected from humans and microorganisms were detected and identified using PCR followed by RLB. Bars indicate 95% confidence intervals calculated with Fisher's exact test.

Of all tested ticks 58% were negative for all microorganisms tested for. 16% were positive for *B. burgdorferi s.l.*, 19% for *Rickettsia *spp., 10% for *Babesia *spp. and 12% for *Ehrlichia*/*Anaplasma *spp.. The overall infection rate with *B. burgdorferi *s.l. was 16% (n = 294, CI 12.1-20.5%), which is significantly lower (p = 0.005) than in the early 90s (24% (n = 521, CI 20.7-28.0%) [32]. Different sub-species of *B. burdorferi *s.l. were found during this study of which *B. afzelii *was the most common one. *Rickettsiae *that were identified to species level were *Rickettsia helvetica *and *Rickettsia monacensis*. All but one Babesia species were identified as *Babesia microti*. The *Ehrlichia/Anaplasma *species identified were mainly *Ehrlichia *sp. schotti variant (recently named "*Candidatus *Neoehrlichia mikurensis"), one tick contained *Anaplasma phagocytophilum *and a last one could not be determined to species level. An overview on the identified microorganisms is given in Table 2. No larvae were found positive for *Babesia *and only one for *Borrelia*. Infection rates of the different life stages were calculated and are presented in figure 1.

**Table 2:** Different tick-borne microorganisms found in ticks collected from humans.

Species	positive ticks
*Borrelia burgdorferi *s.l.	47/297
*B. afzelii*	33
*B. garinii*	6
*B. valaisiana*	1
undetermined	7

*Rickettsia *spp.*	55/297
*R. helvetica*	40
*R. monacensis*	11
undetermined	5

*Ehrlichia/Anaplasma *spp.	33/289
*Ehrlichia *sp. schotti variant	31
*A. phagocytophilum*	1
undetermined	1

*Babesia *spp.	28/297
*B. microti*	27
undetermined	1

Microorganisms in tick lysates were detected and identified by PCR followed by RLB.

*one tick had a double infection with *R. helvetica *and *R. monacensis*

In total 22 study participants reported symptoms of which 14 reported reddening around the tick bite site and 6 reported systemic symptoms and 2 reported both. This corresponds to an absolute risk of 11.4% for developing symptoms and 8.3% and 4.1%, respectively, for local reddening and systemic symptoms after a tick bite. Reddening at the bite site did not show the pattern of erythema migrans in any of the cases. Systemic symptoms included fever (n = 3), malaise (n = 3), fatigue (n = 3), panic attacks (n = 1), muscle pain (n = 1), joint pain (n = 1) or stiffness of the neck (n = 1). Three study participants reported symptoms but did not specify them further. Local reddening occurred and systemic symptoms occurred most frequently in participants bitten by an adult tick (n = 8 and n = 3, respectively) followed by those bitten by nymphs (n = 4 and n = 2) and larvae (n = 0 and n = 1). For the remaining cases life stages of ticks were not determined. Three 171 participants reported no symptoms. Eighty-four percent of the participants reported that the tick had been attached less than 24 hours while 4.7% reported that the tick had been attached for more than 48 hours. The occurrence of symptoms or reddening at the bite site was not correlated with the infectious state of the ticks (Table 3, 4, 5). However, a significant positive correlation (at 95% significance) was found between attachment duration of ticks (≥24 h) and symptoms. This was the case for redness (p = 0.08) and systemic symptoms (p = 0.02) analyzed individually and also when symptoms were analyzed conjointly (p = 0.009).

**Table 3:** Relative risks of developing any symptom correlated to various risk factors.

	Exposed individuals	Any symptom	Relative risk (95% CI)	P value
>24 h	30/193	8/30	3.10 (1.43 - 6.75)	0.0092*
*Borrelia*	43/190	7/43	1.60 (0.70 - 3.66)	0.2841
*Rickettsia*	41/190	6/41	1.36 (0.57 - 3.26)	0.5809
*Ehrlichia*	27/182	4/27	1.44 (0.52 - 3.97)	0.5059
*Babesia*	26/190	2/26	0.63 (0.16 - 2.54)	0.7439
Any infection	98/190	14/98	1.64 (0.72 - 3.73)	0.2625

Relative risks were calculated by dividing incidence rates of exposed by incidence rates of unexposed participants. Confidence intervals and p-values were calculated using Fisher's exact test. P-values below 0.05 (*) were regarded as significant.

**Table 4:** Relative risks of developing redness at the bite site correlated to various risk factors.

	Exposed individuals	Redness on bite site	Relative risk (95% CI)	P value
>24 h	30/193	5/30	2.47 (0.92 - 6.60)	0.0801
*Borrelia*	43/190	5/43	1.55 (0.57 - 4.22)	0.3647
*Rickettsia*	41/190	3/41	0.84 (0.25 - 2.80)	1
*Ehrlichia*	27/182	2/27	0.96 (0.23 - 4.04)	1
*Babesia*	26/190	2/26	0.90 (0.22 - 3.74)	1
Any infection	98/190	10/98	1.56 (0.59 - 4.13)	0.4382

**Table 5:** Relative risks of developing systemic symptoms correlated to various risk factors.

	Exposed individuals	Systemic symptoms	Relative risk (95% CI)	P value
>24 h	30/193	4/30	5.43 (1.44 - 20.54)*	0.0215*
*Borrelia*	43/190	2/43	1.14 (0.24 - 5.44)	1
*Rickettsia*	41/190	3/41	2.18 (0.54 - 8.75)	0.3729
*Ehrlichia*	27/182	2/27	2.30 (0.47 - 11.24)	0.2779
*Babesia*	26/190	1/26	0.90 (0.12 - 7.03)	1
Any infection	98/190	5/98	1.56 (0.38 - 6.36)	0.7218

**Figure 2:**
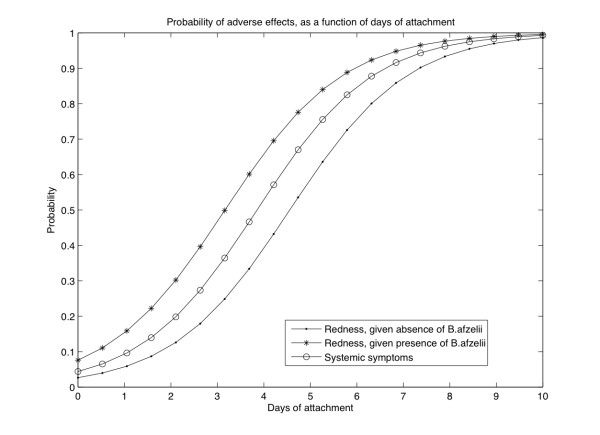
**Probability of adverse effects after a tick bite**. Described as a function of days of attachment and accounting for the presence/absence of *B. afzelii*. The formula describing this relationship can be found in the results section.

Fisher's exact test were significant (p < 0.30) at the 70% level for local redness with attachment duration of more than 24 h, more than 48 h, more than two tick bites and *B. afzelii*, for systemic symptoms with >24 h, *Ehrlichia *spp. and *B. garinii *infection of the tick and for any symptom with >24 h, >48 h, any infection of the tick, *B. afzelii *and *Borrelia burgdorferi s.l*.. Logistic regression with these factors and categorical data yielded no significant result for systemic symptoms. For redness, or any symptoms the following significant regression was found,

$$R=\frac{e^{0.79d}e^{1.1A}}{36.6+e^{0.79d}e^{1.1A}},$$

$$G=\frac{e^{0.8d}}{21.8+e^{0.8d}},$$

where *R *is the probability of redness at *d *days of tick presence and *A *indicates absence ('0') or presence ('1') of *B. afzelii*. *G *is the probability of systemic symptoms depending again on the number of days *d*. Logistic regression indicated the presence of one *B. burgdorferi *sub-species, *B. afzelii*, to be associated with local reddening (Figure 2). For example, 4 days of *B. afzelii *presence yields a probability of redness of 66%.

## Discussion

Tick bites may cause serious and lasting effects due to transmitted pathogens. Symptoms, especially localized ones, can also be due to reaction towards the tick itself. During this study several risk factors for developing localized and systemic symptoms were investigated. Ticks were tested for four different groups of micro-organisms. Statistical analysis showed none of the micro-organisms to be significantly associated to systemic symptoms but logistic regression indicated that *B. afzelii *might be associated with local reddening. In this study, only DNA of microorganisms was detected and identified in the ticks. This does not proof the presence of viable microorganisms neither does it tell something about their pathogenicity to humans. Therefore some of the PCR positive ticks might not have carried infectious agents at all and this could contribute to the absence of disease cases during this study. Future studies should aim to investigate whether these microorganisms, especially the less well studied Rickettsiaceae and *Babesia *species, are an emerging risk to public health.

Tick attachment duration was found to be strongly associated with an increased risk for developing localized reddening other than erythema migrans at the bite site as well as systemic symptoms. This association is most probably due to a direct response towards the tick or substances it secreted into the wound. Ticks secrete a complex mixture of compounds, some of which are potentially toxic, and which accumulate during the feeding process. Castelli *et al. *[36] described local reactions as result of tick bites. These included nodules, erythema and alopecia. A longer attachment period is likely to increase the risk of developing localized or systemic symptoms due to excreted tick proteins and also due to pathogens [37]. Timely removal of a tick is therefore a major factor to reduce transmission of potentially toxic tick excretions and tick-borne pathogens and our study supports that it significantly lowers the risk of developing symptoms.

Many microorganisms that do not live symbiotically in ticks need vertebrate hosts to maintain themselves in a tick population. However, transovarial transmission can contribute more or less to the maintenance of microorganisms in ticks, which seems to play a minor role for *Ehrlichia/Anaplasma *and a major role for *Rickettsia *spp.. The prevalence of these bacteria in larvae was already 8.1% and 26%, respectively. For *Rickettsia *spp. the prevalence was similar in adults, nymphs and larvae. This was observed in an earlier study as well and indicates that *Rickettsiae *are transmitted transovarially at a high degree and therefore rely only a little on vertebrate hosts [5]. It was observed that the prevalence of *B. burgdorferi s.l. *in ticks decreased significantly from 1989/1993 to 2004/2008. This seems to contradict findings that Lyme borreliosis is on the rise in the Netherlands [3,4]. However, an increase in disease incidence is more likely due to an increased exposure of people to infected ticks. This can also be caused by a change in recreational behaviour or in the number of ticks present in the environment. Although these factors are difficult to measure and have not been measured during this study it seems likely that these and not an increase in infection rate are responsible for an increase in Lyme borreliosis incidence.

## Conclusion

In our study, the overall risk of developing symptoms after a tick bite is 11.4% and most of these symptoms are restricted to local reactions. The risk of contracting symptoms of Lyme borreliosis after a single tick bite, even if the tick is infested with potential pathogens, is lower than 1%. Based upon the data collected in this study none of the participants developed symptomatic rickettsiosis, babesiosis or ehrlichiosis. This means that the risk of contracting overt symptoms one of these diseases was lower than 0.5% in this study population. The study shows that prompt removal of ticks reduces the risk of developing symptoms after a tick bite. Thorough checking for ticks together with appropriate clothing, tick avoiding behavior and use of insect repellents, is therefore the most powerful measure to prevent tick-borne diseases. Although the risk of developing symptoms after a tick bite is very low, timely removal of the tick is of an essence and this message should be promoted more clearly and with emphasis to the public.

## Competing interests

The authors declare that they have no competing interests.

## Authors' contributions

JJ and AB designed the study, JJ collected data and ticks, ETK, MF and AB performed lab tests and developed new methodology. AS and AH analyzed data and performed statistical analysis. JG supervised part of study. HS and JR wrote initial drafts and HS and ETK wrote the final manuscript. All authors read and approved the final manuscript.

## References

[B1] Girschick HJ Morbach H Tappe D Treatment of Lyme borreliosis Arthritis Res Ther 2009 11 258 10.1186/ar2853 20067594 PMC3003502

[B2] Smith R Takkinen J Lyme borreliosis: Europe-wide coordinated surveillance and action needed? Euro Surveill 2006 11 E060622 060621 10.2807/esw.11.25.02977-en 16819127

[B3] Hofhuis A Giessen Jvd Borgsteede F Wielinga P Notermans D Pelt Wv Lyme borreliosis in the Netherlands: strong increase in GP consultations and hospital admissions in past 10 years Euro Surveill 2006 11 16819128 10.2807/esw.11.25.02978-en

[B4] Hofhuis A Harms MG Giessen JWBvd Sprong H Notermans DW Pelt Wv Ziekte van Lyme in Nederland 1994-2009: Aantal huisartsconsulten blijft toenemen. Is voorlichting en curatief beleid genoeg? Infectieziekten Bulletin 2010 21

[B5] Sprong H Wielinga PR Fonville M Reusken C Brandenburg AH Borgsteede F Gaasenbeek C van der Giessen JW Ixodes ricinus ticks are reservoir hosts for Rickettsia helvetica and potentially carry flea-borne Rickettsia species Parasit Vectors 2009 2 41 10.1186/1756-3305-2-41 19732416 PMC2743653

[B6] Nielsen H Fournier PE Pedersen IS Krarup H Ejlertsen T Raoult D Serological and molecular evidence of Rickettsia helvetica in Denmark Scand J Infect Dis 2004 36 559 563 10.1080/00365540410020776 15370666

[B7] Fournier PE Allombert C Supputamongkol Y Caruso G Brouqui P Raoult D Aneruptive fever associated with antibodies to Rickettsia helvetica in Europe and Thailand J Clin Microbiol 2004 42 816 818 10.1128/JCM.42.2.816-818.2004 14766859 PMC344501

[B8] Nilsson K Septicaemia with Rickettsia helvetica in a patient with acute febrile illness, rash and myasthenia J Infect 2009 58 79 82 10.1016/j.jinf.2008.06.005 18649945

[B9] Nilsson K Lindquist O Pahlson C Association of Rickettsia helvetica with chronic perimyocarditis in sudden cardiac death Lancet 1999 354 1169 1173 10.1016/S0140-6736(99)04093-3 10513711

[B10] Cinco M Luzzati R Mascioli M Floris R Brouqui P Serological evidence of Rickettsia infections in forestry rangers in north-eastern Italy Clin Microbiol Infect 2006 12 493 495 10.1111/j.1469-0691.2006.01385.x 16643531

[B11] Nilsson K Pahlson C Lukinius A Eriksson L Nilsson L Lindquist O Presence of Rickettsia helvetica in granulomatous tissue from patients with sarcoidosis J Infect Dis 2002 185 1128 1138 10.1086/339962 11930323

[B12] Hildebrandt A Hunfeld KP Baier M Krumbholz A Sachse S Lorenzen T Kiehntopf M Fricke HJ Straube E First confirmed autochthonous case of human Babesia microti infection in Europe Eur J Clin Microbiol Infect Dis 2007 26 595 601 10.1007/s10096-007-0333-1 17587072

[B13] Hunfeld KP Hildebrandt A Gray JS Babesiosis: recent insights into an ancient disease Int J Parasitol 2008 38 1219 1237 10.1016/j.ijpara.2008.03.001 18440005

[B14] Martinot M Zadeh MM Hansmann Y Grawey I Christmann D Aguillon S Jouglin M Chauvin A De Briel D Babesiosis in immunocompetent patients, Europe Emerg Infect Dis 2011 17 114 116 10.3201/eid1701.100737 21192869 PMC3204631

[B15] Kjemtrup AM Conrad PA Human babesiosis: an emerging tick-borne disease Int J Parasitol 2000 30 1323 1337 10.1016/S0020-7519(00)00137-5 11113258

[B16] Doudier B Olano J Parola P Brouqui P Factors contributing to emergence of Ehrlichia and Anaplasma spp. as human pathogens Vet Parasitol 2010 167 149 154 10.1016/j.vetpar.2009.09.016 19836890

[B17] van Dobbenburgh A van Dam AP Fikrig E Human granulocytic ehrlichiosis in western Europe N Engl J Med 1999 340 1214 1216 10.1056/NEJM199904153401517 10206853

[B18] Valenzuela JG Exploring tick saliva: from biochemistry to 'sialomes' and functional genomics Parasitology 2004 129 Suppl S83 94 10.1017/S0031182004005189 15938506

[B19] Steen NA Barker SC Alewood PF Proteins in the saliva of the Ixodida (ticks): pharmacological features and biological significance Toxicon 2006 47 1 20 10.1016/j.toxicon.2005.09.010 16364387

[B20] Francischetti IM Sa-Nunes A Mans BJ Santos IM Ribeiro JM The role of saliva in tick feeding Front Biosci 2009 14 2051 2088 10.2741/3363 19273185 PMC2785505

[B21] Fernandez-Soto P Davila I Laffond E Lorente F Encinas-Grandes A Perez-Sanchez R Tick-bite-induced anaphylaxis in Spain Ann Trop Med Parasitol 2001 95 97 103 10.1080/00034980020035960 11235559

[B22] Acero S Blanco R Bartolome B Anaphylaxis due to a tick bite Allergy 2003 58 824 825 10.1034/j.1398-9995.2003.00211.x 12859569

[B23] Beaudouin E Kanny G Guerin B Guerin L Plenat F Moneret-Vautrin DA Unusual manifestations of hypersensitivity after a tick bite: report of two cases Ann Allergy Asthma Immunol 1997 79 43 46 10.1016/S1081-1206(10)63082-7 9236498

[B24] Lane RS Piesman J Burgdorfer W Lyme borreliosis: relation of its causative agent to its vectors and hosts in North America and Europe Annu Rev Entomol 1991 36 587 609 10.1146/annurev.en.36.010191.003103 2006870

[B25] Piesman J Mather TN Sinsky RJ Spielman A Duration of tick attachment and Borrelia burgdorferi transmission J Clin Microbiol 1987 25 557 558 3571459 10.1128/jcm.25.3.557-558.1987 PMC265989

[B26] Wang G Ojaimi C Wu H Saksenberg V Iyer R Liveris D McClain SA Wormser GP Schwartz I Disease severity in a murine model of lyme borreliosis is associated with the genotype of the infecting Borrelia burgdorferi sensu stricto strain J Infect Dis 2002 186 782 791 10.1086/343043 12198612 PMC2773673

[B27] Sood SK Salzman MB Johnson BJ Happ CM Feig K Carmody L Rubin LG Hilton E Piesman J Duration of tick attachment as a predictor of the risk of Lyme disease in an area in which Lyme disease is endemic J Infect Dis 1997 175 996 999 10.1086/514009 9086168

[B28] Costello CM Steere AC Pinkerton RE Feder HM Jr A prospective study of tick bites in an endemic area for Lyme disease Conn Med 1989 53 338 340 2758821

[B29] Nilsson K Lukinius A Pahlson C Moron C Hajem N Olsson B Lindquist O Evidence of Rickettsia spp. infection in Sweden: a clinical, ultrastructural and serological study Apmis 2005 113 126 134 10.1111/j.1600-0463.2005.apm1130206.x 15723687

[B30] Bjoersdorff A Wittesjo B Berglun J Massung RF Eliasson I Human granulocytic ehrlichiosis as a common cause of tick-associated fever in Southeast Sweden: report from a prospective clinical study Scand J Infect Dis 2002 34 187 191 10.1080/00365540110080061 12030391

[B31] Jacobs JJ Noordhoek GT Brouwers JM Wielinga PR Jacobs JP Brandenburg AH [Small risk of developing Lyme borreliosis following a tick bite on Ameland: research in a general practice] Ned Tijdschr Geneeskd 2008 152 2022 2026 18825891

[B32] Rijpkema S Nieuwenhuijs J Franssen FF Jongejan F Infection rates of Borrelia burgdorferi in different instars of Ixodes ricinus ticks from the Dutch North Sea Island of Ameland Exp Appl Acarol 1994 18 531 542 10.1007/BF00058936 7628258

[B33] Jongejan F Rijpkema S [Borrelia burgdorferi from Ixodes ricinus ticks on Ameland] Tijdschr Diergeneeskd 1989 114 1195 1197 2688191

[B34] Tijsse-Klasen E Fonville M Reimerink JH Spitzen-van der Sluijs A Sprong H Role of sand lizards in the ecology of Lyme and other tick-borne diseases in the Netherlands Parasit Vectors 2010 3 42 10.1186/1756-3305-3-42 20470386 PMC2890652

[B35] Dohoo I Martin W Stryhn H Veterinary Epidemiological Research 2007 Charlottetown: AVC Inc

[B36] Castelli E Caputo V Morello V Tomasino RM Local reactions to tick bites Am J Dermatopathol 2008 30 241 248 10.1097/DAD.0b013e3181676b60 18496425

[B37] Dana AN Diagnosis and treatment of tick infestation and tick-borne diseases with cutaneous manifestations Dermatol Ther 2009 22 293 326 10.1111/j.1529-8019.2009.01244.x 19580576

[B38] Wielinga PR Gaasenbeek C Fonville M de Boer A de Vries A Dimmers W Akkerhuis Op Jagers G Schouls LM Borgsteede F van der Giessen JW Longitudinal analysis of tick densities and Borrelia, Anaplasma, and Ehrlichia infections of Ixodes ricinus ticks in different habitat areas in The Netherlands Appl Environ Microbiol 2006 72 7594 7601 10.1128/AEM.01851-06 17028227 PMC1694262

[B39] Rijpkema SG Molkenboer MJ Schouls LM Jongejan F Schellekens JF Simultaneous detection and genotyping of three genomic groups of Borrelia burgdorferi sensu lato in Dutch Ixodes ricinus ticks by characterization of the amplified intergenic spacer region between 5S and 23S rRNA genes J Clin Microbiol 1995 33 3091 3095 8586679 10.1128/jcm.33.12.3091-3095.1995 PMC228650

[B40] Alekseev AN Dubinina HV Van De Pol I Schouls LM Identification of Ehrlichia spp. and Borrelia burgdorferi in Ixodes ticks in the Baltic regions of Russia J Clin Microbiol 2001 39 2237 2242 10.1128/JCM.39.6.2237-2242.2001 11376063 PMC88117

[B41] Christova I Van De Pol J Yazar S Velo E Schouls L Identification of Borrelia burgdorferi sensu lato, Anaplasma and Ehrlichia species, and spotted fever group Rickettsiae in ticks from Southeastern Europe Eur J Clin Microbiol Infect Dis 2003 22 535 542 10.1007/s10096-003-0988-1 12938010

[B42] Schouls LM Van De Pol I Rijpkema SG Schot CS Detection and identification of Ehrlichia, Borrelia burgdorferi sensu lato, and Bartonella species in Dutch Ixodes ricinus ticks J Clin Microbiol 1999 37 2215 2222 10364588 10.1128/jcm.37.7.2215-2222.1999 PMC85121

[B43] Bergmans AM Groothedde JW Schellekens JF van Embden JD Ossewaarde JM Schouls LM Etiology of cat scratch disease: comparison of polymerase chain reaction detection of Bartonella (formerly Rochalimaea) and Afipia felis DNA with serology and skin tests J Infect Dis 1995 171 916 923 7535830 10.1093/infdis/171.4.916

[B44] Wielinga PR Fonville M Sprong H Gaasenbeek C Borgsteede F Giessen JW Persistent Detection of Babesia EU1 and Babesia microti in Ixodes ricinus in The Netherlands During a 5-Year Surveillance: 2003-2007 Vector Borne Zoonotic Dis 2008 18759637 10.1089/vbz.2008.0047

[B45] Gubbels JM de Vos AP van der Weide M Viseras J Schouls LM de Vries E Jongejan F Simultaneous detection of bovine Theileria and Babesia species by reverse line blot hybridization J Clin Microbiol 1999 37 1782 1789 10325324 10.1128/jcm.37.6.1782-1789.1999 PMC84950

